# Image Descriptors for Weakly Annotated Histopathological Breast Cancer Data

**DOI:** 10.3389/fdgth.2020.572671

**Published:** 2020-12-07

**Authors:** Panagiotis Stanitsas, Anoop Cherian, Vassilios Morellas, Resha Tejpaul, Nikolaos Papanikolopoulos, Alexander Truskinovsky

**Affiliations:** 1 Department of Computer Science and Engineering, University of Minnesota, Minneapolis, MN, United States,; 2 Australian Center for Robotic Vision, Australian National University, Canberra, ACT, Australia,; 3 Department of Pathology & Laboratory Medicine, Roswell Park Cancer Institute, Buffalo, NY, United States

**Keywords:** connected health for breast cancer, image descriptors, annotated data, histopathological data, connected health and computer vision

## Abstract

**Introduction:**

Cancerous Tissue Recognition (CTR) methodologies are continuously integrating advancements at the forefront of machine learning and computer vision, providing a variety of inference schemes for histopathological data. Histopathological data, in most cases, come in the form of high-resolution images, and thus methodologies operating at the patch level are more computationally attractive. Such methodologies capitalize on pixel level annotations (tissue delineations) from expert pathologists, which are then used to derive labels at the patch level. In this work, we envision a digital connected health system that augments the capabilities of the clinicians by providing powerful feature descriptors that may describe malignant regions.

**Material and Methods:**

We start with a patch level descriptor, termed Covariance-Kernel Descriptor (CKD), capable of compactly describing tissue architectures associated with carcinomas. To leverage the recognition capability of the CKDs to larger slide regions, we resort to a multiple instance learning framework. In that direction, we derive the Weakly Annotated Image Descriptor (WAID) as the parameters of classifier decision boundaries in a Multiple Instance Learning framework. The WAID is computed on bags of patches corresponding to larger image regions for which binary labels (malignant vs. benign) are provided, thus obviating the necessity for tissue delineations.

**Results:**

The CKD was seen to outperform all the considered descriptors, reaching classification accuracy (ACC) of 92.83%. and area under the curve (AUC) of 0.98. The CKD captures higher order correlations between features and was shown to achieve superior performance against a large collection of computer vision features on a private breast cancer dataset. The WAID outperform all other descriptors on the Breast Cancer Histopathological database (BreakHis) where correctly classified malignant (CCM) instances reached 91.27 and 92.00% at the patient and image level, respectively, without resorting to a deep learning scheme achieves state-of-the-art performance.

**Discussion:**

Our proposed derivation of the CKD and WAID can help medical experts accomplish their work accurately and faster than the current state-of-the-art.

## INTRODUCTION

About one in eight U.S. women (about 12%) will develop invasive breast cancer over the course of her lifetime^1^. Even though there is a widespread adoption of mammography, interpretation of these images remains challenging. Some of the fundamental morphological characteristics of malignant tumors includes (i) an increased number of cell nuclei per unit area, (ii) increased size of the nuclei, (iii) the nuclei staining darker than those of benign cells (nuclear hyperchromasia), (iv) greater than normal variability in the size and shape of nuclei, and (v) irregular nuclear contours. Therefore, the number, irregularity, and contrast of edges are all expected to increase in malignant tumors compared with benign tissues as noted by Basavanhally et al. (1) and Irshad et al. (2). The diagnostic questions pathologists face depend on the clinical situation and the required characteristics for determining whether a lesion is cancerous. The use of Computer Aided Diagnosis (CAD) schemes can better assist medical experts with their everyday tasks in determining whether a lesion is cancerous or not, the geometric characteristics of the location of the tumor, size, and its relation to the surgical margins with anatomic and histological landmarks.

Cancerous tissue recognition (CTR) from histopathological data is a particularly challenging task since it requires a close examination of tissue slides from suspected regions under a microscope which can be time-consuming hence constraining the number of cases pathologists can handle daily.

An automated identification of the regions that are highly likely to be cancerous can assist experts in finding them among the surrounding tissues efficiently, resulting in faster diagnosis. This is a part of a larger vision in digital connected health that will enable clinicians not matter where they are located to provide more informed assessments and decision-making than the current state-of-the-art.

In order to be trained effectively, most available cancerous tissue recognition (CTR) schemes require pixel level annotations, collected in the form of tissue delineations from expert pathologists [e.g., Sirinukunwattana et al. (3), Spanhol et al. (4), Xu et al. (5), and Xu et al. (6)], which are then used to produce labels at the patch level. Nevertheless, collecting such delineations is error prone and depends on individual experts’ judgment toward identifying accurate transition boundaries between healthy and tumorous tissues. In contrast, relaxing the requirement for such tight tissue delineations and instead asking for annotations only at the bounding box (or whole slide) level can significantly reduce the effort from the experts. Similar considerations have appeared in the medical image analysis literature [e.g., Bejnordi et al. (7), Dundar et al. (8), Xu et al. (5), and Xu et al. (6)]. However, to the best of our knowledge, such studies have not looked at weakly-supervised inference from the perspective of representation learning, which is the primary contribution of this work. In this work, we propose a framework for training cancerous tissue recognition (CTR) schemes in the presence of weakly annotated data to expedite the analysis of Hematoxylin & Eosin (H & E)-stained tissue samples.

We propose a two-step framework for recognition in breast cancer data. Our key insight comes from the process by which the tissue slides are stained, specifically, the Hematoxylin & Eosin (H&E) staining scheme. This process gives unique color and texture to the tissue samples, and our approach is to derive a feature descriptor that leverages on these image properties. First, we derive the Covariance-Kernel Descriptor (CKD), a patch level descriptor that compactly describes tissue architectures associated with malignant areas and achieves superior performance on the problem of Cancerous Tissue Recognition (CTR) against a diverse collection of image descriptors including deep learning derived features. The origins of the Covariance-Kernel Descriptor (CKD) in his area can be traced in a previous work from our group by Stanitsas et al. (9). Second, we devise the Weakly Annotated Image Descriptor (WAID), an image descriptor geared toward larger slide regions that capitalizes on the covariance-kernel descriptor (CKD). The weakly annotated image descriptor (WAID) provides inference on larger image regions, while uplifting the requirement for pixel level annotations.

## MATERIALS AND METHODS

### Data Description

#### Fully Annotated Breast Cancer Database (FABCD)

**FIGURE 1 |:**
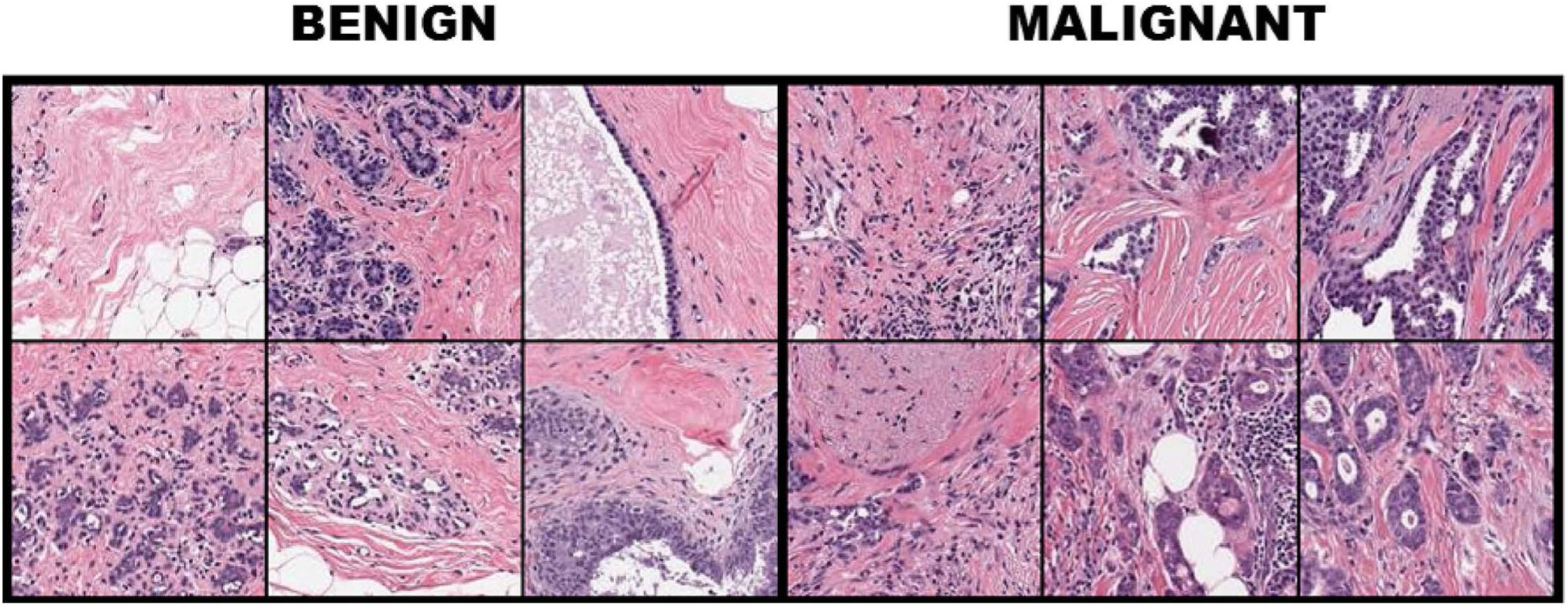
Breast tissue H&E stained patches from 12 samples of the breast cancer dataset with the first three columns illustrating benign cases, while columns 4–6 depict image patches associated with carcinomas.

For FABCD, tissue samples collected are Hematoxylin & Eosin (H&E) stained (10), followed by high-resolution (10K × 9K pixels) scans of tissue sections taken at x50 magnification on a digital slide scanner. Medical experts (surgical pathologists) were responsible for providing annotations corresponding to the malignant and benign image regions. The annotated regions are then divided into smaller disjoint patches of 150 × 150 pixels. Twenty-one annotated images of carcinomas and 19 images of benign tissue taken from 21 patients were combined toward constructing the FABCD. Binary class labels are assigned to each of the image patches in Figure 1. That is, those patches for which more than 80% of the pixels correspond to carcinomas are treated as the positive class, while patches in the negative class are devoid of any cancerous regions.

#### Breast Cancer Histopathological Database (BreakHis)

**FIGURE 2 |:**
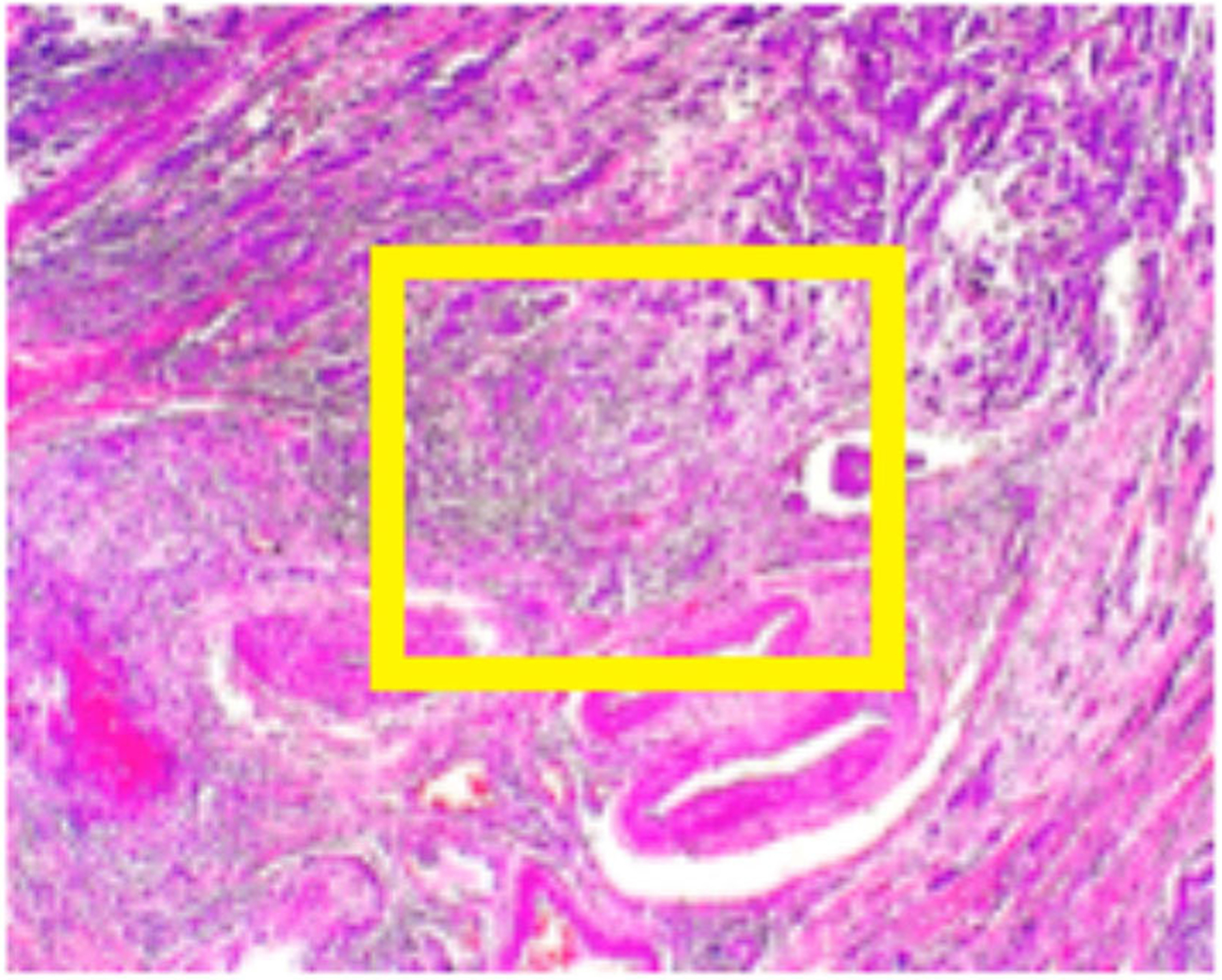
BreakHis sample at 40x magnification for a malignant segment tissue with a yellow bounding box indicating the approximate location of the malignant tissue (for visualization purposes) surrounded by healthy tissue.

BreakHis (11) contains data from 82 patients at four different digital magnifications (40X, 100X, 200X, and 400X). For every magnification level approximately 2,000 H&E-stained tissue slides are collected of size 700 × 460 pixels, while binary labels (benign vs. malignant) and ordinal (four types of malignant and four types of benign) are provided. The magnification of 40x is aligned with the objectives of this study. Medical expert is requested to provide images in the form of bounding boxes surrounding suspicious regions of the whole slide as shown in Figure 2.

#### Covariance-Kernel Descriptors (CKD)

In this work, we compute the region covariance descriptors (RCDs) as proposed by Porikli et al. (12) over a set of features extracted from every pixel in the image patch. In their basic form, RCDs (denoted *C*_*z*_) by Tuzel et al. (13) are generated as described in Equation (1), where *fi* ∈ *R*^*d*^, are *d*-dimensional features extracted from each pixel *i ϵ* {1,2, ⋯, *N*} of an image patch **z**, and *μ* is the mean feature given by $\mu =\frac{1}{N}{\;\sum }_{i=1}^{N}f_{i}$.

(1) $$C_{z}=\frac{1}{\left(N-1\right)}\sum_{i=1}^{N}\left(f_{i}-\mu \right){\left(f_{i}-\mu \right)}^{T}.$$

We consider a 5-dimensional RCD consisting of the normalized intensities of the three channels **R**, **G**, and **B** of a color patch combined with first-order gradient information along the *x* and *y* axis, as denoted by ${\text{Gr}}_{\text{i}}^{\text{x}}$ and ${\text{Gr}}_{\text{i}}^{\text{y}}$ respectively. That is, our *f*_*i*_ has the following form (for pixel *i* in the image patch):

(2) $$f_{i}={\left[\begin{array}{c} {\text{R}}_{\text{i}}{\text{ G}}_{\text{i}}{\text{ B}}_{\text{i}}{\text{ Gr}}_{\text{i}}^{\text{x}}{\text{ Gr}}_{\text{i}}^{\text{y}} \end{array}\right]}^{T}.$$

Covariance-kernel descriptors (CKDs) are computed as the fusion of the Region Covariance Descriptors (RCDs) (12) and Normalized Color Histograms (NCHs) (in conjunction with the work in (14)) that are used to reveal information uncovered by the Hematoxylin & Eosin (H&E) staining. Toward deriving the NCH, for a given patch, we computed color histograms consisting of 256 bins each for the R, G, and B color channels; this histogram is normalized to sum to one and concatenated to form a 768-dimensional feature descriptor for the respective patch. RCDs compute the feature correlations at the pixel level (local) in a patch and in that way capture texture and shape in the patch implicitly. In contrast, NCH represents global color information at the patch’s vicinity. The combination of both global and local information captures complementary cues for recognition which are essential. However, rather than concatenating the three histograms, as in the case of NCH, we combine them to formulate a matrix ***H*** *ϵ R*^3×*b*^, where each row corresponds to the *b*-bin histogram on a channel and enables us to capture global color correlations *via* the modality ***HH***^***T***^. In that way, for an image patch ***z***, the CKD is computed in the form of a compact block diagonal symmetric positive definite (SPD) matrix descriptor that contains in its first block the RCD denoted by *C*_*z*_, while the second block captures the correlations between the histograms computed on the three color channels of the image patch, as formally defined in Definition 1.

**Definition 1**. (Covariance-Kernel descriptor). The Covariance-Kernel descriptor, for an image patch **z** is defined as:

(3) $$D_{z}=\left[\begin{array}{ll} C_{z}+\epsilon \;I_{d1} & 0_{d1}\\ 0_{d2} & H_{z}H_{z}^{T}+\epsilon \;I_{d2} \end{array}\right]$$

where *ϵ* > 0 is a very small constant, *d*_1_ and *d*_2_ are equal to the dimensionality of *C*_*z*_ and $H_{z}H_{z}^{T}$ respectively, 0_*d*1_ and 0_*d*2_ are square zero matrices of dimension *d*_1_ and *d*_2_ respectively, while *I*_*d*1_ and *I*_*d*2_ are the identity matrices of dimension *d*_1_ and *d*_2_ respectively.

Given that the 3 × 3 histogram correlation matrix $H_{z}H_{z}^{T}+$ is positive definite, and thus a valid Mercer kernel, we further improve its representational power by computing the correlations *via* a kernel function. That is, suppose *h*_*c*_ ∈ *R*^*b*^ denotes a histogram vector (where ∈ {*R, G, B*}), then we replace the Gram matrix $H_{z}H_{z}^{T}$ in (3) by a kernel matrix ***K***_***z***_ defined by *K* (*h*_*c*_1, *h*_*c*_2) = *φ* (*h*_*c*_1)^*T*^ *φ* (*h*_*c*_2) for *c*1, *c*2 ∈ {*R, G, B*} and a feature map *φ*. For our task, the linear kernel performed the best among the *χ*^2^, Radial Basis Function (RBF) and polynomial kernels.

**Theorem 1** (positive definiteness of the CKD). For an image patch ***z***, its corresponding CKD, ***D***_***z***_ is an SPD matrix. That is:

(4) $$v^{T}D_{z}v>0,\forall v\in {\mathbb{R}}_{d}-\left\{0_{d}\right\}$$

Proof: Let $v={\left[\begin{array}{c} v_{C}^{T}\;v_{H}^{T} \end{array}\right]}^{T}$, where $v_{C}\in {\mathbb{R}}^{d}$, $v_{H}\in {\mathbb{R}}^{d2}$ with *d*_1_ and *d*_2_ *cor*responding to the size of ***C***_***z***_ *and* $H_{z}H_{z}^{T}$ respectively. That way

(5) $$v^{T}D_{z}v=\left[\begin{array}{cc} v_{C}^{T} & v_{H}^{T} \end{array}\right]\left[\begin{array}{ll} C_{z}+\epsilon \;I_{d1} & 0_{d1}\\ 0_{d2} & H_{z}H_{z}^{T}+\epsilon \;I_{d2} \end{array}\right]{\left[\begin{array}{cc} v_{C}^{T} & v_{H}^{T} \end{array}\right]}^{T}\;=v_{C}^{T}\left(C_{z}+\epsilon \;I_{d1}\right)v_{C}+v_{H}^{T}\left(H_{z}H_{z}^{T}+\epsilon \;I_{d2}\right)\;v_{H}$$

Since *C*_*z*_ ⪰ 0 and ***H***_***z***_***H***^***T***^ ⪰ 0 they both become SPD *via* a small additive perturbation on their diagonal. Thus, both terms of the summation become positive, validating that *v*^*T*^*D*_*z*_*v* > 0.

#### Geometry of CKD

While the CKD already uses rich non-linearities to capture useful higher-order cues in the data, the positive definiteness structure, as shown in Theorem 1, further allows the use of non-linear geometries to significantly improve the recognition performance. That is, instead of using a Euclidean distance to measure the similarity between two SPD matrices, a non-linear measure is used which governs the geometry of the space of these matrices.

In our experiments, we adopt two such measures for efficiently computing similarities between SPD matrices, namely (i) the Log-Euclidean Riemannian metric, and the recently introduced (ii) Jensen-Bregman Logdet Divergence. Of these two, (i) also defines a Riemannian geometry to the space of SPD matrices and is a geodesic distance, while (ii) defines an information geometry-based similarity measure.

First, the Log-Euclidean Riemannian Metric (LERM) Arsigny et al. (15) is described in Equation (6) for a pair of CKDs *D*_*i*_ and *D*_*j*_. In Riemannian geometry, the set of symmetric matrices forms a tangent space for the Riemannian manifold of SPD matrices, and the space of symmetric matrices is isomorphic to the Euclidean space. Thus, taking the matrix logarithm embeds the SPD matrices into a flat tangent space of symmetric matrices on which the usual Euclidean distance can be used for similarity computations. The Euclidean distance is:

(6) $$\text{LERM }\left({\text{D}}_{\text{i}},{\text{D}}_{\text{j}}\right):={\left\|\text{log }\left({\text{D}}_{\text{i}}\right)-\text{log }\left({\text{D}}_{\text{j}}\right)\right\|}_{F}$$

where *Log* (.) is the matrix logarithm and ∥ .∥_*F*_ is the Frobenius norm.

Second, the Jensen-Bregman LogDet Divergence (JBLD), first proposed by Cherian et al. (16), is also considered for similarity computations. In contrast to LERM, JBLD retains the rich non-linear geometry of the space of SPD matrices, and at the same time is computationally cheaper as the matrix logarithms are replaced by matrix determinants which can be computed efficiently *via* Cholesky factorization.

(7) $$\text{JBLD }\left({\text{D}}_{\text{i}},{\text{D}}_{\text{j}}\right):={\left[\text{log }\left|\frac{{\text{D}}_{\text{i}}+{\text{D}}_{\text{j}}}{2}\right|-\frac{1}{2}\text{log }\left|{\text{D}}_{\text{i}}{\text{D}}_{\text{j}}\right|\right]}^{1/2}$$

where |*A*| is the determinant of SPD matrix *A*.

### Weakly Annotated Image Descriptor (WAID)

In an effort to broaden the recognition abilities of the CKD to larger tissue regions (and potentially whole slides) we resort to Multiple Instance Learning (MIL) (17). In the MIL setting, we only need to know if there is at least one patch that is benign or malignant in a whole slide, usually called a bag, and the MIL formulation needs to incorporate the task of inferring which instance in the bag belongs to the concerned class. Similar considerations were presented in Wang and Cherian (18) for activity recognition in a deep learning framework. Our scheme differs from the work in Wang and Cherian (19) in that WAID is computed on symmetric positive definite (SPD) matrices whose geometry is different from descriptors used in action recognition in Wang and Cherian (19). The proposed Weakly Annotated Image Descriptor (WAID) uses the MIL setup to provide annotations at the bag level thus relaxing the requisite for tissue delineations and is devised as the parameters of decision boundaries between positive bags and negative bags.

To formalize the derivation of the WAID, we let a weakly annotated image *i* (malignant or benign disease) be denoted by $Z_{i}^{+}$. Performing a random sub-sampling of *m* patches of size *n × n* for each image allows for expressing $Z_{i}^{+}$ as the set $\left\{Z_{i}^{+}[1],Z_{i}^{+}[2],\ldots ,Z_{i}^{+}[m]\right\}$. For a bag to be characterized as positive the requirement is that at least one of the contained instances is positive which in this work translates to containing tumor tissue (benign disease or malignant). In contrast, for a bag to be negative all instances need to be negative, which is equivalent to containing neither benign diseased nor malignant patches. To achieve this, we contrast our positive bags against negative bags of background classes. In particular, we devise three strategies for populating negative bags with instances namely, (i) random noise images, (ii) images from a surrogate texture recognition dataset [Mallikarjuna et al. KTH (20)] and, (iii) patches depicting healthy regions from H&E breast tissue. In that way, we let ${Ƶ}_{j}^{-}$ denote a negative bag, containing $\left\{Z_{j}^{-}[1],Z_{j}^{-}[2],\ldots ,Z_{j}^{-}[M]\right\}$ instances derived from a background class. Prior to adopting the MIL machinery to our problem, it is required that we provide a compact description of the patches organized in bags; for this task, we employ the CKD. The CKD is a mapping from the space of image patches to that of SPD matrices as $f:{\mathbb{R}}^{n\times n}\to S_{++}^{d}$. In that way, we express ${\tilde{Ƶ}}_{i}^{+}$ and ${\tilde{Ƶ}}_{i}^{-}$ as the sets $\left\{D_{1}^{+},D_{2}^{+},\ldots ,D_{m}^{+}\right\}$ and $\left\{D_{1}^{-},D_{2}^{-},\ldots ,D_{m}^{-}\right\}$ respectively.

The WAID is devised based on variants of the SparseMIL (21) framework, originally designed for applications which exhibit sparse positive bags (containing few positive instances); such an application is image region classification. In particular, we compute the WAID by solving an SVM objective. In that way, for every image *i* we identify the optimal decision boundary parametrized by **w**_**i**_ and **b**_**i**_ such that the percentage of classifiable positive instances is ≥*η*.

Given a positive bag ${\tilde{Ƶ}}_{j}^{-}$ and at least one negative bag ${Ƶ}_{i}^{+}$ we aggregate their instances in {*D*_1_, *D*_2_, …, *D*_*N*_} along with their associated instance level labels {*y*_1_, *y*_2_, …, *y*_*N*_} such that *y*_*i*_ = +1 if $D_{i}\in {Ƶ}_{i}^{+}$ and −1 otherwise; *N* here is the total number of instances in the considered bags. For all *D*_*i*_’s we compute their matrix logarithm [*via* the operator Log (·)] which is equivalent to projecting the CKDs to the tangent to the cone plane which was shown to have a positive effect on similarity computations for SPD matrices (15) as described for LERM [refer to (6)].

Toward allowing for non-linear classification boundaries in the SVM model, we compute explicit feature maps **Ψ** (·) which linearly approximate the Jensen-Shannon’s homogenous kernel based on the work by Vedaldi and Zisserman (22). This allows for the computation of a linear SVM on the feature maps while encapsulating important non-linearities for separating instances belonging to the positive bag from instances in the negative bag(s). As a result, the parameters of the classification boundary are easily captured in **w**_**i**_, which for the non-linearized case becomes less trivial. Then *χ*^2^ and the intersection kernel were also considered with the Jensen-Shannon’s kernel achieving the highest performance among them. For simplifying the notation we let **d**_*i*_ denote the vector resulting from concatenating the columns of *Log* (*D*_*i*_). The classifier is, in that way, computed in a kernel Hilbert space H for which the inner product is defined as 〈**Ψ** (*d*_**i**_), **Ψ** (**d**_**j**_)〉_*H*_ = **Ψ** (**d**_**i**_)^***T***^ **Ψ** (d_**j**_)

With the above notation, we propose our multiple-instance max-margin WAID learning as:

(8) $$\underset{w_{i},b_{i},\xi }{\text{min }}{\left\|w_{i}\right\|}_{2}^{2}+C\sum_{k=1}^{N}\xi_{k}\text{ subject to}\;y_{k}\left(w_{i}^{T}\text{Ψ}\left(d_{k}\right)+b_{i}\right)\geq 1-\xi_{k},\forall k\in \left\{1,\ldots ,N\right\}\;\xi_{k}>0,\;\forall k\in \;\left\{1,\ldots ,N\right\}\;\frac{\left|y_{i}^{+}\right|}{\left|{Ƶ}_{i}^{+}\right|}\geq \eta$$

where $y_{i}^{+}$ denotes the set of instances that receive a positive label by the trained ∞-SVM (23), that has three important components, namely (i) the WAID descriptor defined by the pair (w, b), (ii) the class labels y that is −1 for all instances in the negative bag, however is either +1 or −1 depending on whether the optimization decides the instance in the positive bag is positive or negative, and (iii) a proportionality constraint that says that we know a proportion defined by *η* of the positive bag has positive instances. The hyper-parameter η needs to be decided *via* cross validation or from experience.

To accommodate constraints that are difficult to cater to, we incorporate slack variables denoted by *ξ*_k_ to handle the non-separability of the samples as defined below.

(9) $$\text{sign }\left(w\frac{T}{i}\psi \left(d_{l}\right)+b_{i}\right)=+1,\forall d_{l}\in y_{i}^{+}.$$

**FIGURE 3 |:**
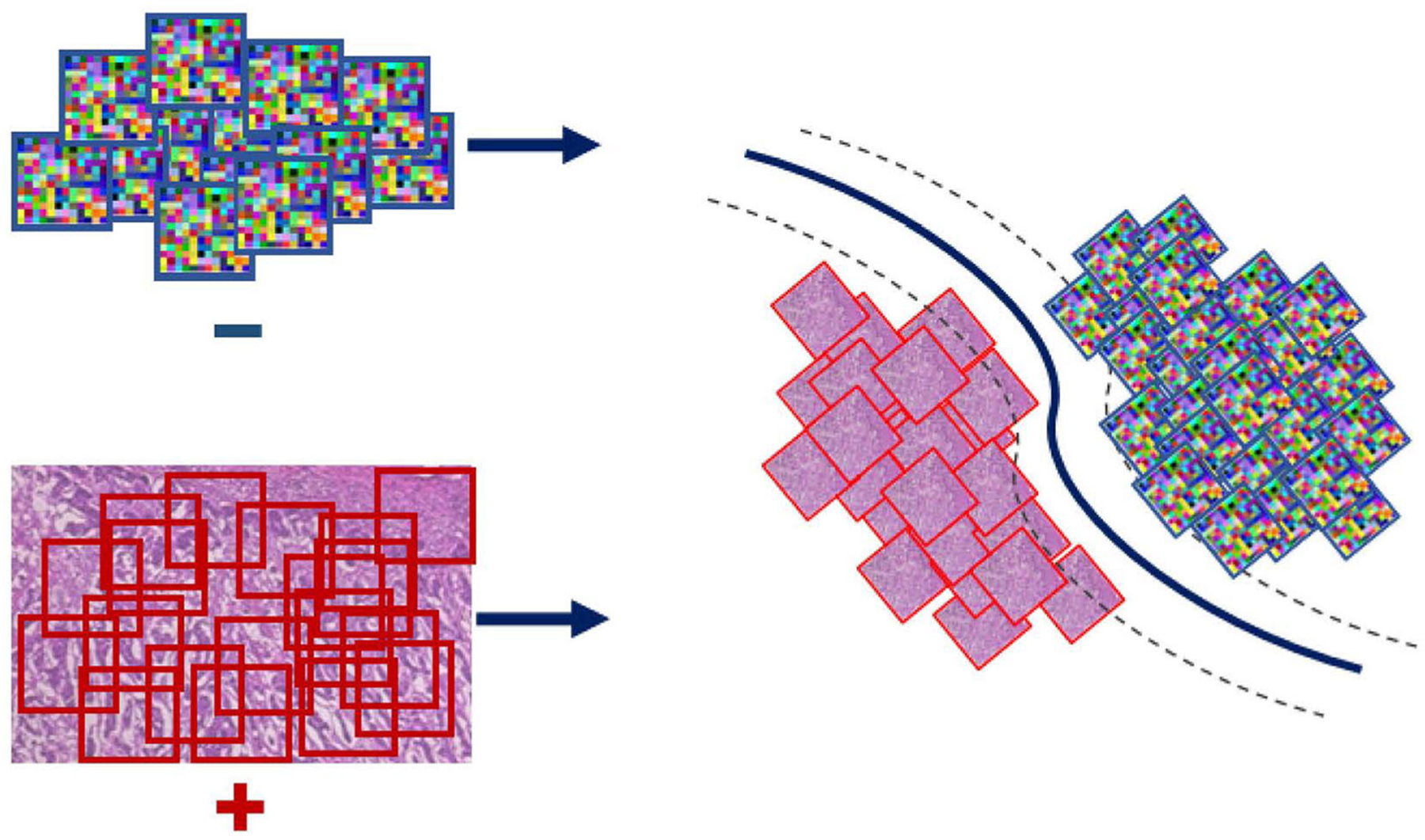
WAID computation. Images are sub-sampled, and descriptors are computed for every derived patch. The WAID is computed as the vector containing the parameters of an SVM model computed in a multiple instance learning framework.

Even though the conventional SVM part of the formulation is convex, and thus can be solved efficiently *via* standard optimization machinery, the *η*-constraint makes it combinatorial. An important observation for solving this problem is the effect of the regularization parameter *C* on the objective; larger values of *C* penalize more steeply misclassified instances. Toward satisfying the *η*-constraint, computed on the ratio $\frac{\left|y_{i}^{+}\right|}{\left|{Ƶ}_{i}^{+}\right|}$, the SVM objective is iteratively solved for increasing values of *C*. In particular, starting with a small value for the parameter *C* we retrieve a solution and check if the *η*-constraint is satisfied based on that. In the case that the condition is not satisfied, the parameter *C* is rescaled to a larger value making the formulation less tolerant to misclassifications and thus steering it toward making more positive predictions. In the case that the condition is met, the SVM objective is solved for that value of *C* and the parameters of the classifier (W_I_ and *b*_*I*_) are extracted and used to form the WAID. More formally, the WAID (Figure 3) for an image *I* is presented in Definition 2.

**Definition 2** (WAID). The Weakly Annotated Image Descriptor for an image I is defined as:

(10) $$W_{I}={\left|w_{I}^{T}\;b_{I}\right|}^{T}$$

Once the WAID is computed for every sample in a given set of images, standard machine learning techniques are implemented toward learning based on the patterns uncovered by the descriptor.

**FIGURE 4 |:**
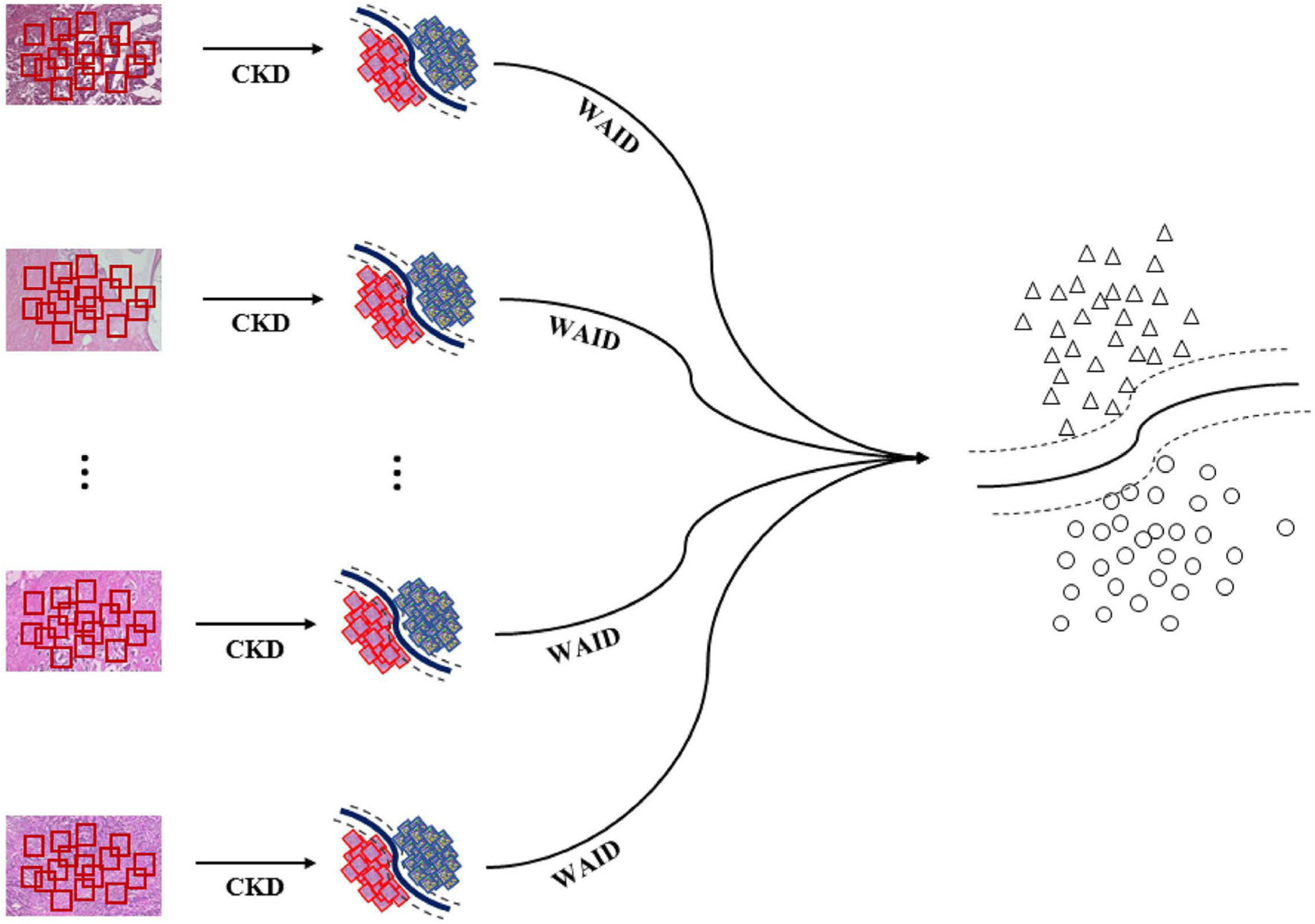
Weakly annotated data processing. First, images are sub-sampled and CDKs are computed on every patch. Second, the WAID is computed for every group of patches. Finally, an SVM model is computed for classifying malignant images.

The overall pipeline for processing the aforementioned benchmark is illustrated in Figure 4. First, images are sub-sampled and for the generated patches CKD descriptors are computed. Second, for every group of patches the WAID is computed then an SVM model is computed on the resulting WAID representations.

## RESULTS

In this section, we present our experiments on the two databases described in the methods. First, we present a thorough evaluation of the CKD on the FABCD against a very large collection of image descriptors computed at the patch level. Following that, we evaluate the WAID on the BreakHis dataset against Multiple Instance Learning (MIL) alternatives as well as schemes that have been previously proposed for providing inference on the dataset.

### FABCD

**FIGURE 5 |:**
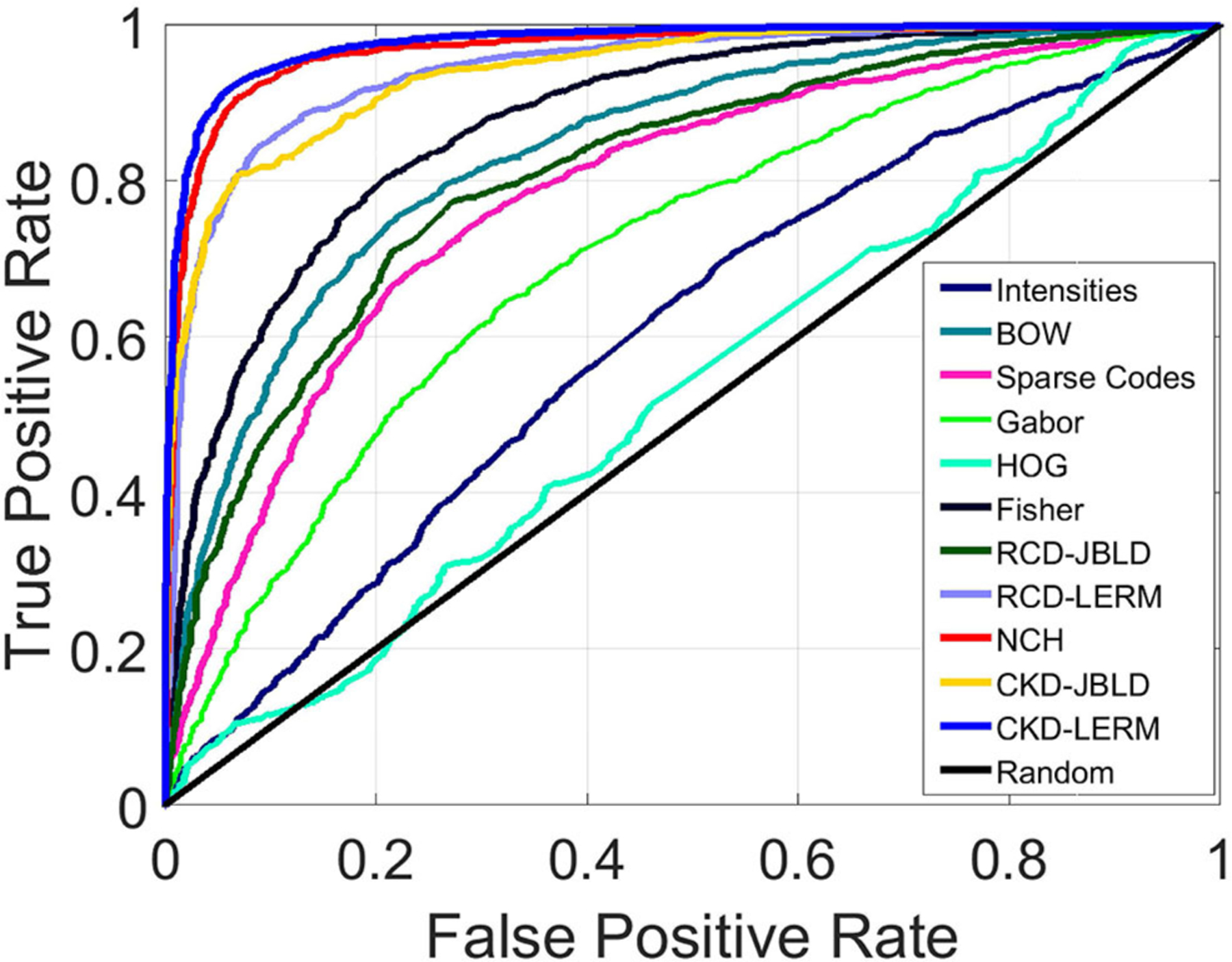
ROC curve for intermediate level descriptors on FABCD (Best if viewed in color).

We present comparisons using SVMs, while for all the learned models we evaluate the classification performance using two different metrics, namely (i) classification accuracy (ACC), and (ii) the Area Under the Curve (AUC) computed from Receiver Operating Characteristic (ROC) curves in a 10-fold cross-validation. For RCDs and CKDs, we use Radial Basis Function (RBF) Mercer kernels based on the LERM and the JBLD measures stated in the Supplementary Material. For the rest of the tested descriptors, a collection of different kernels and parameter configurations were tested. In particular, the tested kernels were linear, polynomial, RBF, and Sigmoid. In Figure 5 we can see that for almost all features represented, linear kernels achieved the highest performance and were used to report our results. The only exception is the kernel utilized for the Gabor features which is a polynomial kernel of third degree.

Figure 5 above presents the resulting ROC curves for the conducted experiments. Among edge-based descriptors, Fisher Vectors (FVs) appear to achieve the highest accuracy as well as AUC, reaching accuracy of 79.66%. The NCH IV-A outperformed all the edge-based descriptors achieving a high accuracy value of 91.63%, accompanied by very high AUC. RCDs reported accuracy that was on par with the performance of the NCHs. Finally, the CKD was seen to outperform all the considered descriptors, reaching ACC of 92.83% and AUC of 0.98. Table 1 below aggregates the results obtained on FABCD for all the described intermediate level descriptors in terms of ACC and AUC, as computed for the extracted ROC curves.

#### Comparisons Against CNNs

Even though CNN based representations would require patch level annotations for their crafting, we believe that presenting comparisons against popular CNN topologies is very important. It should be noted though, that in the general weakly supervised setup patch level annotations are not necessarily available. Since we have data limited to a few thousand samples, we fine-tuned two popular CNN topologies with weights learned on the 1M image database of the ILSVRC challenge. For this study, we established a comparison against the Alexnet (24) and VGG16 (25) topologies. We compare against well-known CNN models that are often found to be generically useful for a variety of tasks. However, our experiments show that in small-data regimes, training such large topologies leads to overfitting and thus reduced performance in comparison to feature representations that are tailored to the task, as is the case with our proposed CKD descriptor.

The results of this section are delivered in the form of ACC and AUC in a 10-fold validation setup in Table 2. The CKD when combined with LE similarities is seen to outperform the Alexnet topology which achieved ACC of 89.23%. Finally, the VGG-16 was able to outperform the CKD achieving ACC of 93.91% and AUC of 0.99.

### BreakHis

#### Parameter Tuning

**FIGURE 6 |:**
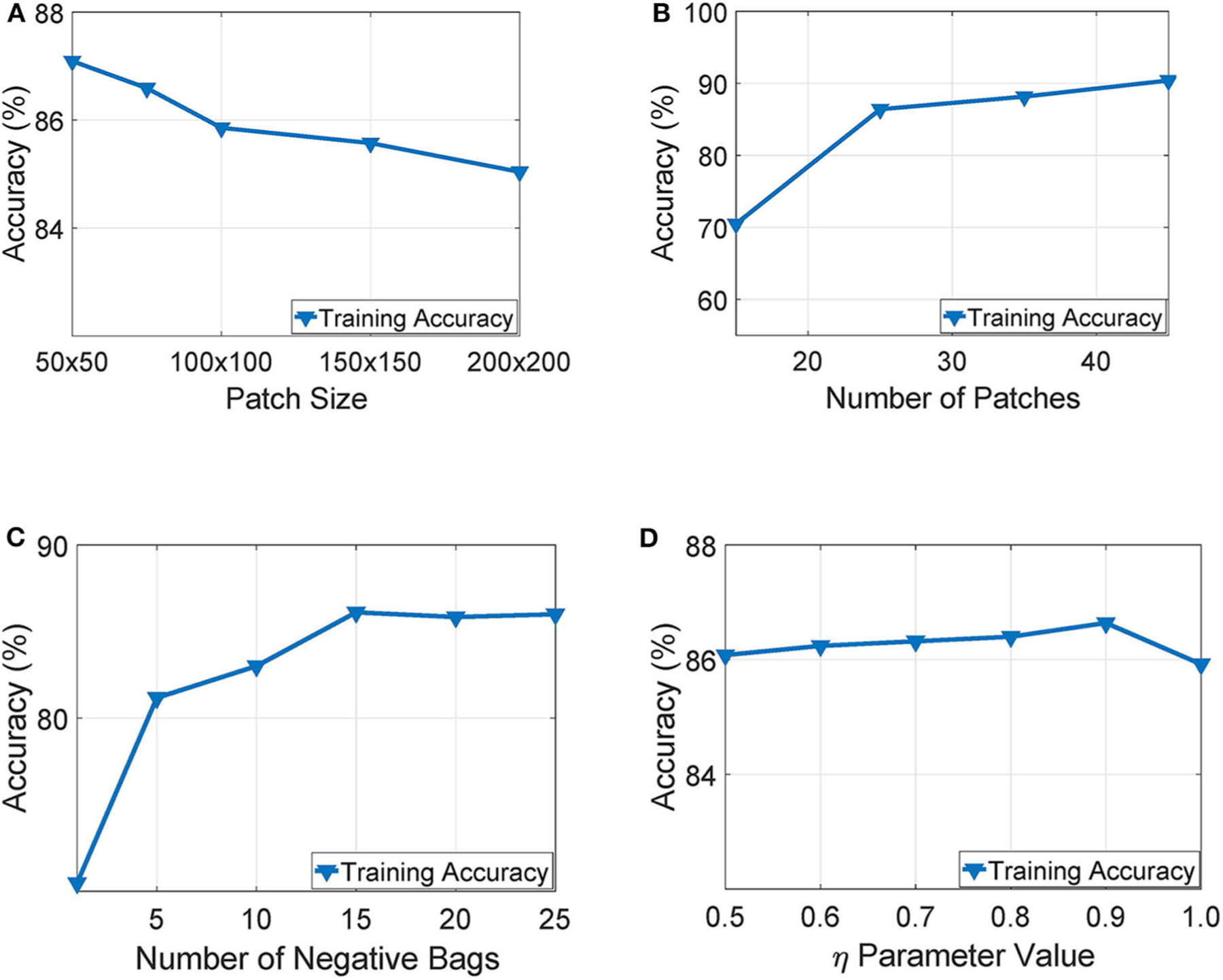
Parameter exploration for, **(A)** patch size, **(B)** number of patches, **(C)** number of negative bags and, **(D)** η parameter.

Our experimentation indicated that working with tissue slides collected at 40x magnification level and patches of size 50 × 50 yielded the highest training accuracy as also illustrated in Figure 6A. Similarly, Figure 6B presents a parameter exploration with respect to the number of patches sub-sampled from the initial slide. We found that sampling 25 patches yielded the optimal recognition accuracy since it balances between training accuracy and over-fitting. Furthermore, as illustrated in Figure 6C, we can see that working with more than 15 negative bags did not improve the performance of the WAID. Finally, Figure 6D depicts the performance of the devised scheme against different values of the parameter η. We select η = 0.9.

**FIGURE 7 |:**
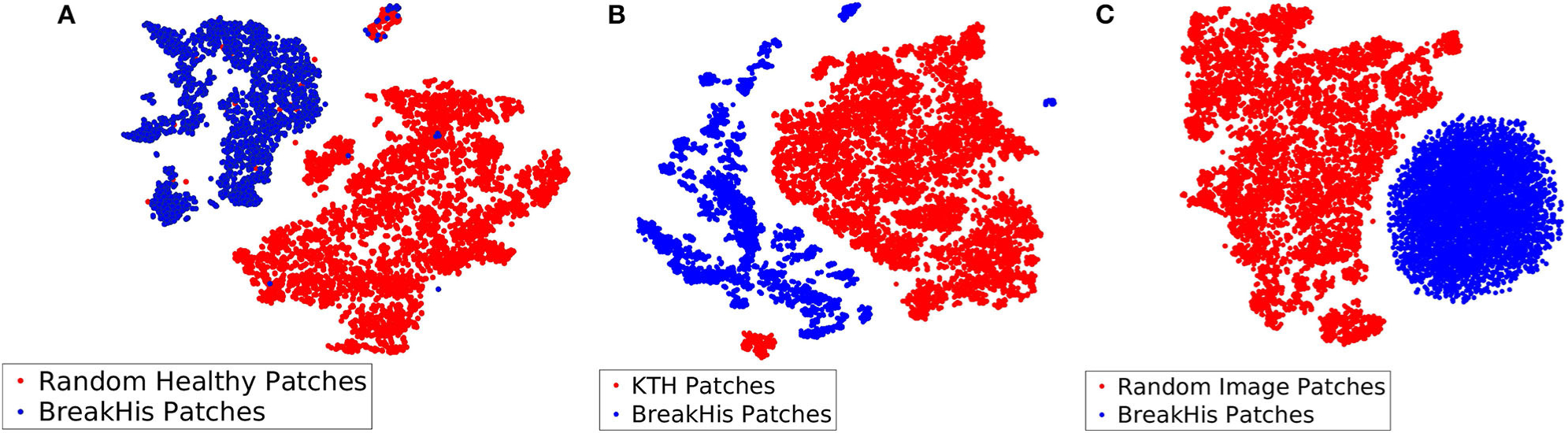
Low dimensional embeddings of CKDs on sub-sampled patches of BreakHis images against CKDs computed on **(A)** patches of healthy tissue, **(B)** KTH patches, and **(C)** random noise images.

Among the three different types of background bags (KTH, healthy tissue, and random noise), we found that working with random noise images yielded the highest accuracy. Low dimensional embeddings by der Maaten and Hinton (26) of CKDs computed on instances of the aforementioned bags (blue dots) are plotted against CKDs computed on patches of BreakHis (red dots) in Figure 7. For the case of healthy tissue patches, the overall performance was hindered by the risk of steering the decision boundaries around healthy samples since the positive bags also contain instances corresponding to healthy tissue deteriorating the overall performance. This can result in the inaccurate enclosure of the benign or malignant tumor instances as also suggested by Figure 7A. In addition, the KTH database offers a large variability in the types of contained textures resulting in a less firm cluster formation when plotted against CKDs on the histopathological data as also illustrated in Figure 7B. Finally, when working with random images for the background class, as presented in Figure 7C, it resulted in a better separation from the tissue samples which was also imprinted in our results.

#### Comparisons Against MIL Schemes

The comparisons of the WAID against MIL based alternatives and a baseline corresponding to computing a CKD descriptor on the whole image termed Single-CKD (S-CKD) was reported. The results are shown in terms of accuracy and the area under the curve averaged across the 5-folds provided with the benchmark. First, we considered the MIL-SVM Andrews et al. (27) scheme, the Sparse-MIL (21) was included in this set of experiments, since it takes into account the sparse distribution of positive instances in the positive bags which we set to 0.3 (an estimate of the percentage of cancerous tissue against healthy in the image). In both, we used a linear kernel. Third, in a boosting setup we present comparisons against the MIL-Boost (28) and the MCIL-Boost (6) schemes, for which we use 50 weak classifiers. The number of weak classifiers was identified *via* a trial and error process in an effort to control the amount of over-fitting of the model on the training sets. Additionally, for the MCIL-Boost (6) scheme we present results for two and three clusters in the positive bags. The number of clusters in the data was aligned with the characteristics of the dataset according to which samples contain malignant tissue surrounded by healthy tissue and potentially transition areas between the two. Finally, to further motivate the adaptation of a MIL based scheme we present results based on image descriptors computed at the whole view (S-CKD). For S-CKD and the WAID, we use an SVM model with an RBF kernel with *γ* = 0.00025. For the experiments involving the following schemes, namely, (i) MIL-SVM, (ii) SIL and, (iii) Sparse-MIL, we used the MISVM python module distributed in support of Doran and Ray (29). Furthermore, for the MIL-Boost and MCIL-Boost schemes we used the distribution, accompanying the work by Xu et al. (5, 6).

Summarizing the contents of Table 3, we see that MIL-SVM and Sparse MIL achieved the lowest performance achieving an average 0.76 and 0.70 AUC across the five computed folds. Following that, the S-CKD achieved an AUC of 0.83 underlining the necessity of the MIL paradigm. The fusion of boosting and MIL was shown to be sufficient to exceed the three aforementioned baselines, and its performance was exceeded by allowing for multiple clusters in the data through MCIL-Boost. The latter achieved an AUC value of 0.89 accompanied by ACC of 80.13 and 80.14% at the image and patient level, respectively. MCIL-Boost was only outperformed by the proposed WAID which reached an AUC of 0.90 accompanied by ACC of 83.57 and 85.50% at the patient and image level, respectively.

#### Comparisons Against State-of-the-Art Schemes

**FIGURE 8 |:**
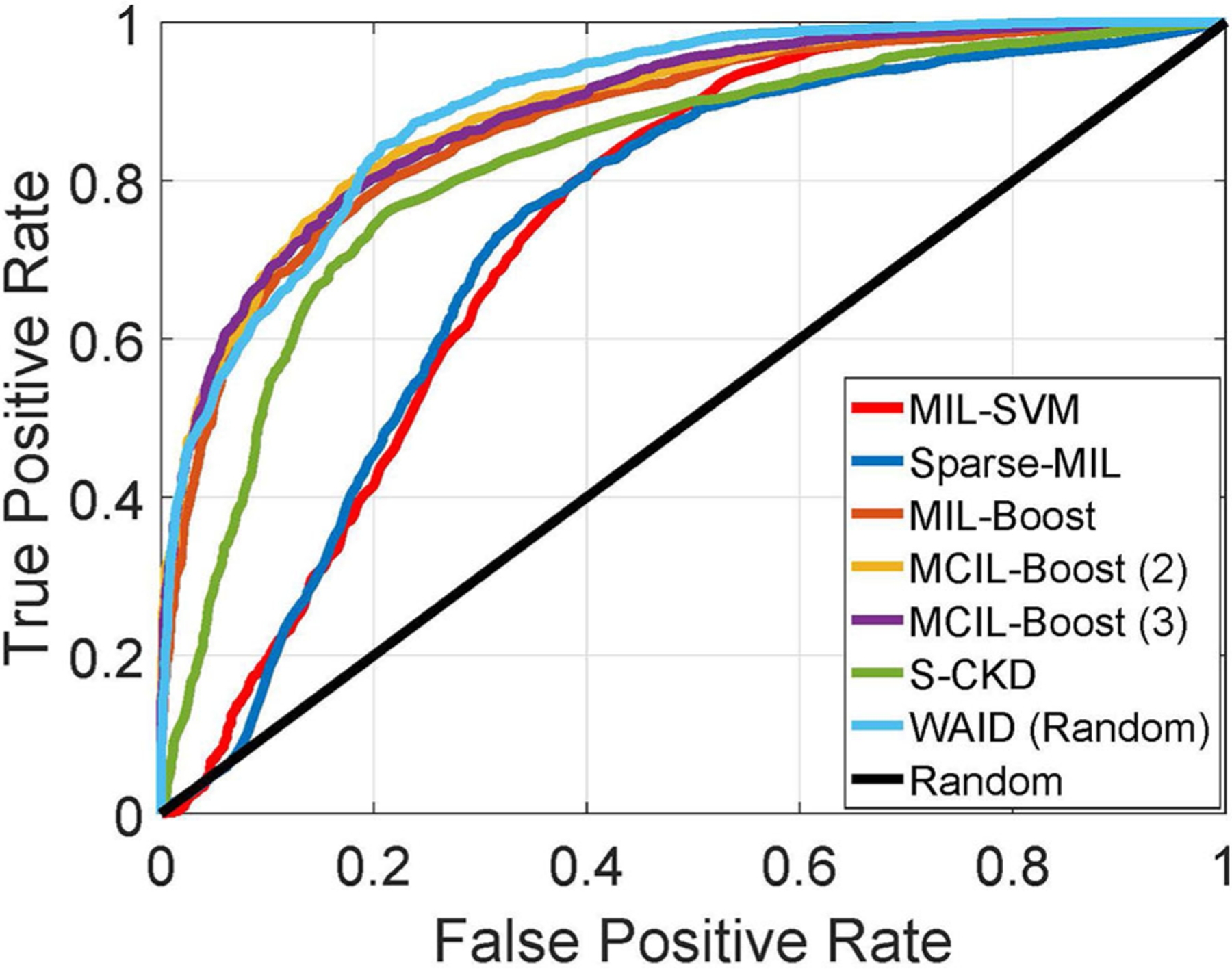
ROC curve for experiments against MIL schemes on BreakHis (Best if viewed in color).

In this section, we establish comparisons against visual learning schemes that have been previously deployed for providing inference on the selected magnification (x40) of the BreakHis dataset. We compare against the work by Spanhol et al. (11) for which Parameter Free Threshold Adjacency Statistics (PFTAS) features (30) were computed and coupled with different classifiers namely, (i) 1-Nearest-Neighbor, (ii) Quadratic Discriminant Analysis, (iii) Random Forests, and (iv) Support vector machines. Comparisons are also established with the work by Spanhol et al. (4) which proposed a CNN based on the (24) topology. Furthermore, we present results against the work by Song et al. (31) that utilized a Fisher Vector based scheme. Finally, a CNN based scheme capitalizing on the GoogleNet topology (32) was presented by Das et al. (33). It should be noted that we are not concerned with fusion rules on the predictions of multiple images as in Spanhol et al. (4) and Das et al. (33), and we focus our evaluation on the predictions at the image and patient levels as reported in the respective studies. In the aforementioned studies, results were presented in the form of Correctly Classified Malignant (CCM) instances, at the slide level as well as the patient level. It should be noted that for the PFTAS based schemes the authors did not provide slide level performance statistics, while for Das et al. (33) the CCM at the patient level is based on majority voting in contrast to averaging as deployed in all other schemes. Figure 8 shows the ROC curve for the experiments.

Table 4 summarizes the results obtained on the BreakHis by the WAID against recently published schemes on this dataset. The proposed framework outperforms existing approaches achieving state-of-the-art performance on BreakHis with its CCM reaching 91.27 and 92.00% at the patient and image level, respectively, without resorting to a deep learning scheme, thus making WAID a computationally attractive and easier to implement alternative.

## DISCUSSION

In this work, we presented a framework for the analysis of histopathological breast cancer data in the presence of weak supervision. The proposed Covariance-Kernel descriptor (CKD) manages to capture higher order correlations between edges and color information (as the result of the staining process) that are very important for the recognition of malignant areas while enclosing them in a compact representation. Although the CKD successfully characterizes tissue architectures at the patch level, its performance deteriorates as the targeted slide regions increase in size. This can be attributed to the fusion of different tissue types in larger slide regions (healthy, benign disease, and malignant regions). To address this shortcoming, while leveraging the recognition capability of the CKD to larger regions of the slide (and potentially the whole slide), we derive an image descriptor in a Multiple Instance Learning (MIL) (17) framework that builds upon the CKDs. The MIL paradigm was selected due to its ability to provide inference for data organized in the form of bags (larger slide regions or whole slides) containing not individually labeled instances (patches). In pursuance of obviating the necessity for pixel level annotations, we propose the weakly annotated image descriptor (WAID) which solely requires weakly annotated samples in the form of binary labels (malignant vs. benign) and is capable of characterizing larger slide regions. Based on the results gathered from the experiments, we concluded that WAID is able to achieve state-of-the-art performance on a database that contains weakly annotated images.

As personalized medicine becomes prevalent, medical experts are faced with high demands to create automation of their most recurrent tasks and for a more complex set of analyses to be done (34). The average patient waits approximately 10 days for a pathology result, which can be critical for some patients when it comes to treatment plans as their safety and health are at risk^2^. Samples containing a large set of data require substantial effort and time from medical experts who have to manually segment the data. With these challenges, it is essential to address real-world medical challenges, solve clinical or public health problems, and recognize patients’ needs (35). An automated model will allow medical diagnosis to be made at a timelier and prompter rate, thereby allowing patients to receive their results earlier which minimizes both anxiety and delayed treatments. Our model does not require an extensive amount of effort from medical experts, hence eliminating human errors. In addition, this allows medical experts to focus their time on treatment plans and patient consultations which will further improve the quality of care patients are to receive. This will not only help to improve the patient’s health outcomes but also enhance the quality of health management.

Some limitations of this study are: (1) the dataset is not large enough, (2) the descriptors may not work for other cancers since they may need different weights, and (3) future studies are needed to validate the model.

## CONCLUSION

In this work, we presented a framework for the analysis of histopathological breast cancer data in the presence of weak supervision. This work was concerned with the derivation of a scheme demanding less annotation effort by medical experts. We initiated our analysis with the derivation of an intermediate image representation (patch level), termed CKD, which outperformed a very large collection of popular computer vision descriptors on a private, fully supervised H&E Breast cancer dataset (FABCD). Following that, we proposed an image descriptor, termed WAID, which was derived in a MIL setup for characterizing larger image regions. WAID achieved state-of-the-art performance on the considered magnification level of the BreakHis both against MIL-based schemes as well as prior methods on the database.

Delays in diagnosing cancer is either from providers simply not consider cancer in their differential diagnosis^3^ or by the waiting time of 10 or more days depending on the workload and skills of the expert to collect complicated analysis of H&E slides^2^. By implementing the derivation of a scheme that demands less annotation effort from medical experts, H&E slides can be read at a faster pace without compromising on accuracy enabling providers to determine the diagnose and treatment plan that will lessen the stress, anxiety, and unwanted burden on their patients. In regard to patients’ well-being, our proposed derivation of the CKD and WAID can help medical experts accomplish their work accurately and faster.

## Supplementary Material

Supplementary Material

## Figures and Tables

**TABLE 1 |:** Experimental results on FABCD.

Features	ACC	AUC
Intensities	57.91%	0.60
HOG	51.86%	0.53
Gabor	65.60%	0.71
Fisher	79.66%	0.88
Sparse codes	72.31%	0.78
BOW	76.46%	0.84
RCD-JBLD	74.26%	0.81
RCD-LE	87.66%	0.94
NCH	91.63%	0.97
CKD-JBLD	85.51%	0.94
**CKD-LE**	**92.83%**	**0.98**

The bold values highlight the best performance.

**TABLE 2 |:** Experimental results on FABCD against CNNs.

Features	ACC	AUC
CNN(AlexNet)	89.23%	0.96
**CNN(VGG-16)**	**93.91%**	**0.99**
CKD-LE	92.83%	0.98

The bold values highlight the best performance.

**TABLE 3 |:** Comparisons against different frameworks for weakly supervised data on BreakHis.

Method	ACC		AUC
	Patient	Image	Image
MIL-SVM (27)	73.24	71.42	0.76
Sparse-MIL (21)	71.53	71.27	0.70
MIL-Boost (28)	79.54	79.68	0.87
MCIL-Boost (6) (c = 2)	80.44	79.73	0.89
MCIL-Boost (6) (c = 3)	80.14	80.13	0.89
S-CKD	77.99	77.40	0.83
WAID (KTH)	84.05	82.02	0.87
WAID (Healthy)	80.63	79.98	0.86
**WAID (Random)**	**85.50**	**83.57**	**0.90**

The bold values highlight the best performance.

**TABLE 4 |:** Comparisons against state-of-the-art on BreakHis.

Method	CCM	
	Patient	Image
PFTAS-1NN (11)	80.90	-
PFTAS-QDA (11)	83.60	-
PFTAS-RF (11)	81.80	-
PFTAS-SVM (11)	81.60	-
CNN-Alexnet (4)	89.60	88.60
Adaptive-Fisher (31)	87.00	90.00
CNN-Googlenet (33)	-	91.26
**WAID (Random)**	**91.27**	**92.00**

The bold values highlight the best performance.

## Footnotes

1 Breast Cancer: Statistics. Available online at: https://www.breastcancer.org/

2 Tests-and-procedures: biopsy. Available online at: www.cancer.net.

3 Available online at: https://www.frontiersin.org/research-topics/13170/use-of-primary-care-datasets-for-high-risk-and-early-cancer-detection
