# Supplementary material for: Image Descriptors for Weakly Annotated Histopathological Breast Cancer Data
Source: Front Digit Health. 2020 Dec 7;2:572671. doi: 10.3389/fdgth.2020.572671 (PMC7749086; doi:10.3389/fdgth.2020.572671)
Supplement: Supplementary file 1 [file Table_1.DOCX]

Supplementary Material

**A. Covariance-Kernel Descriptors**

In this work, we compute RCDs over a set of features extracted from every pixel in the image patch. In their basic form, RCDs (denoted$C_{z}$) are generated as described in Equation (1), where $f_{i}\in R^{d}$, are *d*-dimensional features extracted from each pixel $i \epsilon$ {1*,*2*,*··· *,N*} of an image patch $\boldsymbol{z}$, and $\mu$ is the mean feature given by $\mu=\frac{1}{N} \sum_{i=1}^{N} f_{i}$ .

*Equation 1:* $C_{z}=\frac{1}{\left( N-1 \right)} \sum_{i=1}^{N} \left( f_{i}-\mu\right)\left( f_{i}-\mu\right)^{T}$.

We consider a 5-dimensional RCD consisting of the normalized intensities of the three channels **R**, **G**, and **B** of a color patch combined with first-order gradient information along the *x* and *y* axis, as denoted by $Gr {}_{i}^{x}$and ${Gr}_{i}^{y}$respectively. That is, our$f_{i}$ has the following form (for pixel *i* in the image patch):

*Equation 2:* $f_{i}=\left[ R_{i} G_{i} B_{i} Gr {}_{i}^{x} {Gr}_{i}^{y} \right]^{T}$.

CKDs are computed as the fusion of the RCDs and Normalized Color Histograms (NCHs) that are used to reveal information uncovered by the H&E staining. Towards deriving the NCH for a given patch, we computed color histograms consisting of 256 bins each for the R, G, and B color channels; this histogram is normalized to sum to one and concatenated to form a 768-dimensional feature descriptor for the respective patch.

RCDs compute the feature correlations at the pixel level (local) in a patch and in that way capture texture and shape in the patch implicitly. In contrast, NCH represents global color information at the patch’s vicinity. The combination of both global and local information captures complementary cues for recognition which are essential. However, rather than concatenating the three histograms, as in the case of NCH, we combine them to formulate a matrix $\boldsymbol{H} \epsilon R^{3\times b}$, where each row corresponds to the *b*-bin histogram on a channel and enables us to capture global color correlations via the modality $\boldsymbol{H}\boldsymbol{H}^{\boldsymbol{T}}$**.**

In that way, for an image patch$\boldsymbol{z}$, the CKD is computed in the form of a compact block diagonal SPD matrix descriptor that contains in its first block the RCD denoted by $C_{z}$, while the second block captures the correlations between the histograms computed on the three color channels of the image patch, as formally defined in Definition 1.

**Definition 1**. (Covariance-Kernel descriptor). *The Covariance-Kernel descriptor, for an image patch* $\boldsymbol{z}$ *is defined as:*

*_Equation 3:_* $D_{z}=\left[ \begin{matrix} {C_{z}+ \epsilon{I_{d}}_{1} \atop{0_{d}}_{2}} & {{0_{d}}_{1} \atop H{}_{z}H_{z}^{T}+ \epsilon{I_{d}}_{2}} \end{matrix} \right]$

*where* $\epsilon>0$*is a very small constant,* $d_{1}$ *and* $d_{2}$ *are equal to the dimensionality of* $C_{z}$*and* $H{}_{z}H_{z}^{T}$ *respectively,* ${0_{d}}_{1}$*and*  ${0_{d}}_{2}$*are square zero matrices of dimension* $d_{1}$ *and* $d_{2}$*respectively, while* ${I_{d}}_{1}$*and* ${I_{d}}_{2}$*are the identity matrices of dimension* $d_{1}$ *and* $d_{2}$ *respectively.*

Given that the 3x3 histogram correlation matrix $\boldsymbol{H}{}_{\boldsymbol{z}}\boldsymbol{H}_{\boldsymbol{z}}^{\boldsymbol{T}}\boldsymbol{+}$is positive definite, and thus a valid Mercer kernel, we further improve its representational power by computing the correlations via a kernel function. That is, suppose $h_{c}\in R^{b}$ denotes a histogram vector (where $\in\{R,G,B\}$ ), then we replace the Gram matrix $\boldsymbol{H}{}_{\boldsymbol{z}}\boldsymbol{H}_{\boldsymbol{z}}^{\boldsymbol{T}}$in (3) by a kernel matrix $\boldsymbol{K}_{\boldsymbol{z}}$defined by $K\left( h_{c}1,h_{c}2 \right)=\varphi\left( h_{c}1 \right)^{T}\varphi(h_{c}2)$ for $c1,c2\in\{R,G,B\}$ and a feature map $\varphi$. For our task, the linear kernel performed the best among the *χ*^2^, Radial Basis Function (RBF) and polynomial kernels.

**Theorem 1** (Positive Definiteness of the CKD). *For an image patch*$\boldsymbol{z}$*, its corresponding CKD,* $\boldsymbol{D}_{\boldsymbol{z}}$*is an SPD matrix. That is:*

*Equation 4:*  $v^{T}D_{z}v>0,\forall v\in\mathbb{R}_{d}-\left\{ 0_{d} \right\}$

*Proof: Let* $v=\left[ \begin{matrix} v_{C}^{T} & v_{H}^{T} \end{matrix} \right]^{T},$*where* $v_{C}\in\mathbb{R}^{d1}, v_{H}\in\mathbb{R}^{d2}$ *with* $d_{1}$ *and* $d_{2}$*corresponding to the size of* $\boldsymbol{C}_{\boldsymbol{z}}$*and* $\boldsymbol{H}{}_{\boldsymbol{z}}\boldsymbol{H}_{\boldsymbol{z}}^{\boldsymbol{T}}$*respectively. That way*

*Equation 5:* $v^{T}D_{z}v=\left[ \begin{matrix} v_{C}^{T} & v_{H}^{T} \end{matrix} \right]\left[ \begin{matrix} {C_{z}+ \epsilon{I_{d}}_{1} \atop{0_{d}}_{2}} & {{0_{d}}_{1} \atop H{}_{z}H_{z}^{T}+ \epsilon{I_{d}}_{2}} \end{matrix} \right]\left[ \begin{matrix} v_{C}^{T} & v_{H}^{T} \end{matrix} \right]^{T}$

## $\boldsymbol{=}\boldsymbol{v}_{\boldsymbol{C}}^{\boldsymbol{T}}\left( \boldsymbol{C}_{\boldsymbol{z}}\boldsymbol{+}\boldsymbol{\epsilon}{\boldsymbol{I}_{\boldsymbol{d}}}_{\boldsymbol{1}} \right) \boldsymbol{v}_{\boldsymbol{C}}\boldsymbol{+}\boldsymbol{v}_{\boldsymbol{H}}^{\boldsymbol{T}} \left( \boldsymbol{H}{}_{\boldsymbol{z}}\boldsymbol{H}_{\boldsymbol{z}}^{\boldsymbol{T}}\boldsymbol{+\epsilon}{\boldsymbol{I}_{\boldsymbol{d}}}_{\boldsymbol{2}} \right) \boldsymbol{v}_{\boldsymbol{H}}$_._

*Since*$C_{z}⪰0$ *and* $\boldsymbol{H}_{\boldsymbol{z}}\boldsymbol{H}^{\boldsymbol{T}}⪰0$ *they both become SPD via a small additive perturbation on their diagonal. Thus both terms of the summation become positive, validating that* $v^{T}D_{z}v>0$*.*

***1) Geometry of CKD:*** While the CKD already uses rich non-linearities to capture useful higher-order cues in the data, the positive definiteness structure, as shown in Theorem 1, further allows the use of non-linear geometries to significantly improve the recognition performance. That is, instead of using a Euclidean distance to measure the similarity between two SPD matrices, a non-linear measure is used which governs the geometry of the space of these matrices.

In our experiments, we adopt two such measures for efficiently computing similarities between SPD matrices, namely (i) the Log-Euclidean Riemannian metric, and the recently introduced (ii) Jensen-Bregman Logdet Divergence. Of these two, (i) also defines a Riemannian geometry to the space of SPD matrices and is a geodesic distance, while (ii) defines an information geometry based similarity measure.

First, the Log-Euclidean Riemannian Metric (LERM) is described in Equation 6 for a pair of CKDs $D_{i}$ and$D_{j}$. In Riemannian geometry, the set of symmetric matrices forms a tangent space for the Riemannian manifold of SPD matrices, and the space of symmetric matrices is isomorphic to the Euclidean space. Thus, taking the matrix logarithm embeds the SPD matrices into a flat tangent space of symmetric matrices on which the usual Euclidean distance can be used for similarity computations. The Euclidean distance is:

*_Equation 6:_* $LERM\left( D_{i},D_{j} \right):=\left\| \mathrm{Log}\left( D_{i} \right)-Log\left( D_{j} \right) \right\|_{F}$

where $\mathrm{Log}\left( . \right)$is the matrix logarithm and $\left\| . \right\|_{F}$is the Frobenius norm.

Second, the Jensen-Bregman LogDet Divergence (JBLD), first proposed by Cherian et al. [7], is also considered for similarity computations. In contrast to LERM, JBLD retains the rich non-linear geometry of the space of SPD matrices, and at the same time is computationally cheaper as the matrix logarithms are replaced by matrix determinants which can be computed efficiently via Cholesky factorization.

*Equation 7:* $JBLD (D_{i},D_{j}):={[\log\left| \frac{D_{i}+D_{j}}{2} \right|-\frac{1}{2}\log\left| D_{i}D_{j} \right|]}^{1/2}$

where $\left| A \right|$is the determinant of SPD matrix A.

***B. Weakly Annotated Image Descriptor***

In an effort to broaden the recognition abilities of the CKD to larger tissue regions (and potentially whole slides) we resort to MIL. Following the MIL paradigm, we allow for the data to be organized in bags (larger slide regions), containing instances (patches) on which the CKD is implemented. Further, rather than instance level annotations, in this setup, it suffices to provide annotations at the bag level thus relaxing the requisite for tissue delineations. The proposed WAID, is devised as the parameters of decision boundaries between positive bags and negative bags. Similar considerations were presented in [46] for activity recognition in a deep learning framework. To formalize the derivation of the WAID, we let a weakly annotated image $i$ (malignant or benign disease) be denoted by$Z_{i}^{+}$. Performing a random sub-sampling of $m$ patches of size $n\times n$for each image allows for expressing $Z_{i}^{+}$ as the set${\{Z}_{i}^{+}\left[ 1 \right] ,Z_{i}^{+}\left[ 2 \right],\ldots, Z_{i}^{+}[m]\}$. For a bag to be characterized as positive the requirement is that at least one of the contained instances is positive which in this work translates to containing tumor tissue (benign disease or malignant). In contrast, for a bag to be negative all instances need to be negative, which is equivalent to containing neither benign diseased nor malignant patches. To achieve this, we contrast our positive bags against negative bags of background classes. In particular, we devise three strategies for populating negative bags with instances namely, (i) random noise images, (ii) images from a surrogate texture recognition dataset (KTH [27]) and, (iii) patches depicting healthy regions from H&E breast tissue.

In that way, we let$Ƶ_{j}^{-}$denote a negative bag, containing $\{Z_{j}^{-}\left[ 1 \right], Z_{j}^{-}\left[ 2 \right],\ldots, Z_{j}^{-}[M]\}$ instances derived from a background class. Prior to adopting the MIL machinery to our problem, it is required that we provide a compact description of the patches organized in bags; for this task, we employ the CKD. The CKD is a mapping from the space of image patches to that of SPD matrices as $f: \mathbb{R}^{n\times n}\longrightarrow S_{++}^{d}$. In that way, we express

$\tilde{Ƶ}_{i}^{+}$and $\tilde{Ƶ}_{i}^{-}$ as the sets $\left\{ D_{1}^{+}, D_{2}^{+},\ldots, D_{m}^{+} \right\}$ and $\{D_{1}^{-}, D_{2}^{-},\ldots, D_{m}^{-}\}$ respectively.

The WAID is devised based on variants of the SparseMIL [5] framework, originally designed for applications which exhibit sparse positive bags (containing few positive instances); such an application is image region classification. In particular, we compute the WAID by solving an SVM objective. In that way, for every image $i$ we identify the optimal decision boundary parametrized by $\boldsymbol{w}_{\boldsymbol{i}}$and $\boldsymbol{b}_{\boldsymbol{i}}$such that the percentage of classifiable positive instances is greater than or equal to $\eta$.

Given a positive bag $Ƶ_{i}^{+}$and at least one negative bag $Ƶ_{j}^{-}$ we aggregate their instances in $\left\{ D_{1}, D_{2},\ldots, D_{N} \right\}$along with their associated instance level labels $\left\{ y_{1},y_{2},\ldots, y_{N} \right\}$such that $y_{i}=+1$if $D_{i}\inƵ_{i}^{+}$and $-1$otherwise; $N$here is the total number of instances in the considered bags. For all $D_{i}$’s we compute their matrix logarithm (via the operator Log(·)) which is equivalent to projecting the CKDs to the tangent to the cone plane which was shown to have a positive effect on similarity computations for SPD matrices [2].

Towards allowing for non-linear classification boundaries in the SVM model, we compute explicit feature maps $\boldsymbol{\psi}$ (·) which linearly approximate the Jensen-Shannon’s homogenous kernel based on [44]. This allows for the computation of a linear SVM on the feature maps while encapsulating important nonlinearities for separating instances belonging to the positive bag from instances in the negative bag(s). As a result, the parameters of the classification boundary are easily captured in $\boldsymbol{w}_{\boldsymbol{i}}$, which for the non-linearized case becomes less trivial. Then $\chi^{2}$and the intersection kernel were also considered with the Jensen-Shannon’s kernel achieving the highest performance among them. For simplifying the notation we let **d***_i_* denote the vector resulting from concatenating the columns of $\mathrm{Log}\left( D_{i} \right).$ The classifier is, in that way, computed in a kernel Hilbert space H for which the inner product is defined as $\left\langle\boldsymbol{\psi}\left( d_{\mathbf{i}} \right)\boldsymbol{,}\boldsymbol{\psi}\left( \mathbf{d}_{\mathbf{j}} \right) \right\rangle_{H}=\boldsymbol{\psi}\left( \mathbf{d}_{\mathbf{i}} \right)^{\boldsymbol{T}}\boldsymbol{\psi(}d_{j}\boldsymbol{)}$**.**

*Equation 8:*  ${\min_{w_{i},b_{i},\xi} \left\| w_{i} \right\|}_{2}^{2}+C\sum_{k=1}^{N} \xi_{k}$ subject to

$${y_{k}\left( w_{i}^{T}\Psi\left( d_{k} \right)+b_{i} \right)\geq1-\xi_{k}, \forall k\in\{1,\ldots,N\} \atop{\xi_{k} > 0, \forall k\in\{1,\ldots,N\} \atop\frac{|y_{i}^{+}|}{|Ƶ_{i}^{+}|}\geq\eta}}$$

where $y_{i}^{+}$denotes the set of instances that receive a positive label by the trained SVM model and $\xi_{k}$ are slack variables to handle the non-separability of the samples as defined below.

*Equation 9:* $sign (w\frac{T}{i}\psi\left( d_{l} \right)+b_{i})=+1, \forall d_{l} \in y_{i}^{+}$.

Even though the conventional SVM part of the formulation is convex, and thus can be solved efficiently via standard optimization machinery, the *η*-constraint makes it combinatorial. An important observation for solving this problem is the effect of the regularization parameter *C* on the objective; larger values of *C* penalize more steeply misclassified instances. Towards satisfying the *η*-constraint, computed on the ratio $\frac{\left| y_{i}^{+} \right|}{\left| Ƶ_{i}^{+} \right|}$, the SVM objective is iteratively solved for increasing values of *C*. In particular, starting with a small value for the parameter *C* we retrieve a solution and check if the *η*-constraint is satisfied based on that. In the case that the condition is not satisfied, the parameter *C* is rescaled to a larger value making the formulation less tolerant to mis-classifications and thus steering it towards making more positive predictions. In the case that the condition is met, the SVM objective is solved for that value of *C* and the parameters of the classifier (**W_I_** and *b_I_*) are extracted and used to form the WAID. More formally, the WAID is presented in Definition 2.

**Definition 2** (WAID). *The Weakly Annotated Image Descriptor for an image I is defined as:*

*Equation 10:* $W_{I}=\left| w_{I}^{T} b_{I} \right|^{T}$
